# Inhibition of Aerobic Glycolysis Represses Akt/mTOR/HIF-1α Axis and Restores Tamoxifen Sensitivity in Antiestrogen-Resistant Breast Cancer Cells

**DOI:** 10.1371/journal.pone.0132285

**Published:** 2015-07-09

**Authors:** Yu Mi Woo, Yubin Shin, Eun Ji Lee, Sunyoung Lee, Seung Hun Jeong, Hyun Kyung Kong, Eun Young Park, Hyoung Kyu Kim, Jin Han, Minsun Chang, Jong-Hoon Park

**Affiliations:** 1 Department of Life Systems, Sookmyung Women’s University, 52 Hyochangwon Road, Yongsan-gu, Seoul, Republic of Korea; 2 National Research Laboratory for Mitochondrial Signaling Laboratory, Cardiovascular and Metabolic Disease Center, Department of Physiology, College of Medicine, Department of Health Sciences and Technology, Graduate School, Inje University, Gaegume 2 dong, Busanjin-gu, Busan; 3 Department of Medical and Pharmaceutical Sciences, Sookmyung Women’s University, 52 Hyochangwon Road, Yongsan-gu, Seoul, Republic of Korea, Korea; University of South Alabama, UNITED STATES

## Abstract

Tamoxifen resistance is often observed in the majority of estrogen receptor–positive breast cancers and it remains as a serious clinical problem in breast cancer management. Increased aerobic glycolysis has been proposed as one of the mechanisms for acquired resistance to chemotherapeutic agents in breast cancer cells such as adriamycin. Herein, we report that the glycolysis rates in LCC2 and LCC9—tamoxifen-resistant human breast cancer cell lines derived from MCF7— are higher than those in MCF7S, which is the parent MCF7 subline. Inhibition of key glycolytic enzyme such as hexokinase-2 resulted in cell growth retardation at higher degree in LCC2 and LCC9 than that in MCF7S. This implies that increased aerobic glycolysis even under O_2_-rich conditions, a phenomenon known as the Warburg effect, is closely associated with tamoxifen resistance. We found that HIF-1α is activated via an Akt/mTOR signaling pathway in LCC2 and LCC9 cells without hypoxic condition. Importantly, specific inhibition of hexokinase-2 suppressed the activity of Akt/mTOR/HIF-1α axis in LCC2 and LCC9 cells. In addition, the phosphorylated AMPK which is a negative regulator of mTOR was decreased in LCC2 and LCC9 cells compared to MCF7S. Interestingly, either the inhibition of mTOR activity or increase in AMPK activity induced a reduction in lactate accumulation and cell survival in the LCC2 and LCC9 cells. Taken together, our data provide evidence that development of tamoxifen resistance may be driven by HIF-1α hyperactivation via modulation of Akt/mTOR and/or AMPK signaling pathways. Therefore, we suggest that the HIF-1α hyperactivation is a critical marker of increased aerobic glycolysis in accordance with tamoxifen resistance and thus restoration of aerobic glycolysis may be novel therapeutic target for treatment of tamoxifen-resistant breast cancer.

## Introduction

Most tumors that initially respond to tamoxifen eventually acquire resistance to it in 2 to 5 years and acquired tamoxifen resistance is a critical therapeutic problem [1–3]. Understanding the mechanisms of tamoxifen resistance and devising strategies to overcome drug resistance are urgent tasks for developing more successful endocrine therapies. Tamoxifen is known to change the energy metabolism in ER-positive cells or tissues. One group reported a marked difference in the kinetics of glucose metabolism and the concentration of glucose-derived metabolites between 17β-estradiol- and tamoxifen-treated ER-positive breast cancer cells. It was also observed that treatment with tamoxifen reduced the rate of glycolysis and lactate clearance in MCF7 cells by two-fold in both *in vitro* and *in vivo* models [4–6].

Cancer cells frequently display high rates of aerobic glycolysis to generate ATP even under abundant oxygen, which is a phenomenon known as the Warburg effect [7]. The biochemical and molecular mechanisms underlying the Warburg effect appear to be complex and remain to be defined. However, it is widely accepted that cancer cells predominantly produce energy by glycolysis followed by lactic acid fermentation in the cytosol, rather than by oxidation of pyruvate in the mitochondria, which is considered as an energy metabolism pathway in most normal cells [8,9]. In addition, it has been observed that the levels of lactate as the end products of glycolysis are higher in aggressive cancer cells such as drug-resistant or metastatic cancers [10,11], which implies that the Warburg effect in these cancers may reflect metabolic adaptations associated with the development of resistance to chemotherapeutic agents such as doxorubicin, cytosine arabinoside, taxol, cisplatin, and vincristine [12–16]. These findings strongly implicate the presence of a link between altered energy metabolism—in particular, glycolytic metabolism—and resistance to various chemotherapeutic agents. However, no studies have shown the potential role of glycolytic metabolism in the development of tamoxifen resistance.

In the regulation of aerobic glycolysis, hypoxia-inducible factor (HIF)-1α is known to be a key protein, the activation of which induces the expression of genes that encode glucose transporters and glycolytic enzymes [17,18]. However, it is still unclear how cells switch their metabolism from oxidative phosphorylation to aerobic glycolysis [15]. The HIF-1 complex is composed of α and β subunits that bind to hypoxia-responsive elements (HREs) in the promoter region of HIF-1 target genes. HIF-1α can be activated by genetic loss of the von Hippel-Lindau (VHL) tumor suppressor protein or increased oncogenic signaling pathways including Akt/mTOR pathway even under normoxic conditions [19]. Because HIF-1α mRNA contains polypyrimidine tracts, it is generally thought that PI3K/Akt/mTOR stimulates cap-dependent translation of HIF-1 mRNA through the activation of two downstream targets of mTOR, p70S6K and 4E-BP1. Furthermore, it is known that AMP-activated protein kinase (AMPK), which is activated by an increased AMP/ATP ratio acting as an energy sensor [20], directly or indirectly suppresses mTOR activity to limit protein synthesis [21,22]. However, the role of AMPK in the regulation of glycolysis and function of HIF-1 also remains unclear [23]. The recent study reported that HIF-1 contributes to drug resistance in a wide range of neoplastic cell lines such as gastric cancer cells, oral squamous cell carcinoma cells and fibrosarcoma cells [24]. It has been demonstrated that overexpression of an HIF-1 target gene such as pyruvate dehydrogenase kinase-3 induces resistance to chemotherapeutic agents [15,25]. These previous studies imply that alterations in glucose metabolism associated with HIF-1 may play a critical role in the development of drug resistance. However, HIF-1-mediated drug resistance as well as development of tamoxifen resistance in ERα-positive breast cancer cells is a complicated process and it is dependent upon the tumor type. It is essential to characterize the roles of HIF-1 in drug resistant breast cancer cells in connection with abnormal aerobic glycolysis.

The cell lines LCC2 and LCC9 are reliable models for investigating acquired resistance and they have been generated through exposing MCF7 cells to selective estrogen receptor modulators, such as 4-hydroxytamoxifen (the active metabolite of tamoxifen; 4-OHT) and ICI 182,780, respectively [26,27]. Both LCC2 and LCC9 cell lines are ER positive since they are derived from MCF7 cells, which are designated as MCF7S in this study. LCC2 cells are 4-OHT resistant but sensitive to ICI 182,780; whereas, LCC9 cells are cross-resistant to both 4-OHT and ICI 182,780. These two cell lines were chosen as cell line models to investigate the relationship between altered glycolytic metabolism and tamoxifen resistance in our present study.

Here, we showed that LCC2 and LCC9 cells had increased aerobic glycolytic phenotypes compared to MCF7S cells and that regulation of HIF-1α through mTOR and AMPK signaling may be attributed to altered glycolytic metabolism in tamoxifen-resistant cells. These data provide significant information that would help to unravel the relationship between increased aerobic glycolysis and tamoxifen resistance. It is also a step forward in understanding how to overcome tamoxifen resistance through the inhibition of glycolysis by targeting the Akt/mTOR and AMPK-regulated HIF-1α oncogenic signaling pathways.

## Materials and Methods

### Cell culture and chemicals

Different types of sub-lines derived from the MCF7 human breast cancer cell line, including MCF7S, LCC2, and LCC9, were generous gifts from Dr. Clarke (Lombardi Cancer Center, Georgetown University, Washington DC) [26–28]. All three cell lines express both the ER and the progesterone receptor (PR). The cells were grown in RPMI 1640 supplemented with 10% (v/v) bovine calf serum (BCS) and penicillin (100 units/ml) in a humidified 5% CO_2_ atmosphere at 37°C. All of the cell culture reagents were purchased from WelGENE (Daejeon, South Korea) and BCS was obtained from HyClone Labs (Logan, UT). All other molecular biology reagents and chemicals were obtained from Sigma-Aldrich (St. Louis, MO) unless stated otherwise.

### Drug treatment

For drug treatment, cells were plated in 60 mm diameter dishes at a density of 1.5 × 10^6^ cells/dish and the culture medium was replaced with RPMI 1640 containing 0.1% BCS. An appropriate chemical inhibitor (rapamycin at 30 nM or LY294,002 at 40 μM) was added and incubation was continued for another 16 h in the presence of rapamycin or 4 h in the presence of LY294,002. In another experiment, cells were treated with 1 mM of 5-aminoimidazole-4-carboxamide ribonucleoside (AICAR, Cell Signaling Technology, Danvers, MA) for 1 h.

### Quantification of lactate accumulation

Cells were grown in RPMI medium supplemented with 10% BCS and 0.2% uniformly ^13^C-labeled glucose (^13^C_6_-glucose) for 48 h. Lyophilized cell masses were extracted in 60% (v/v) acetonitrile in water to remove proteins. The lyophilized tricarboxylic acid (TCA) extracts of the cells were dissolved in 0.35 or 0.65 ml of D_2_O containing 30 or 50 nmoles of internal standard DSS (sodium 4,4-dimethyl-4-silapentane-1-sulfonate, deuterated at the pentane moiety), respectively. Quantification of cellular lactate accumulation was performed using two different techniques, nuclear magnetic resonance (NMR) and gas chromatography-mass spectrometry (GC-MS), the results of which were confirmatory and complementary, respectively. One-dimensional (1D) ^1^H NMR spectra of the cellular extracts were acquired at 14.1 Tesla on a Varian Inova 4-channel NMR spectrometer using a 5 mm HCN triple resonance cold probe that was maintained at 20°C and a PRESAT pulse sequence. Most of the secreted lactate was derived from ^13^C-labeled glucose, while the contribution from non-glycolytic sources (^12^C-lactate) was less than 1%. Resonances arising from protons attached to ^12^C and ^13^C (as a pair of ^13^C satellites) were evident in the spectral region for the 3-methyl resonance of lactate. The fine splitting pattern of the ^13^C-lactate satellites was consistent with uniformly ^13^C-labeled lactate (^13^C_3_-lactate). Semi-quantitative observation by NMR was complemented by analyzing the same extract using GC-MS. GC-MS measurements were carried out according to the previously published method [29]. Briefly, following NMR analysis, the same TCA extract was re-equilibrated with H_2_O, lyophilized to remove deuterated water, and then silylated with 1:1 (v:v) acetonitrile:MTBSTFA (*N*-methyl-*N*-[*tert*-butyl-dimethylsilyl]trifluoroacetamide) (Regis Chemical, Morton Grove, IL) by carrying out 3 h of sonication followed by allowing the solution to stand overnight. The solution was directly analyzed using a PolarisQ GC-ion trap MSn (Thermo Scientific, Austin, TX) with an injection volume of 0.5 μl. Metabolites were identified and quantified automatically using Xcalibur software (ThermoFinnigan) based on their GC retention times and mass fragmentation patterns, which were matched against an in-house database and external standards. Lactate production was also assayed in each sample using a lactate assay kit (BioVision, Milpitas, CA) according to the manufacturer’s instructions. Briefly, cells were plated at 1 × 10^4^/well in a 96-well plate and l-(+)-lactate standard was diluted with lactate assay buffer to generate 0, 2, 4, 6, 8, and 10 nmol/well. Fifty microliters of the reaction mix containing lactate probe and enzyme mix was added to each well. The final product was quantified at a wavelength 570 nm using a BioTek Elisa instrument (BioTek Instruments, Winooski, VT).

### Glucose uptake

2-Deoxy-d-[^3^H]glucose (GE Healthcare Life Sciences, Pittsburgh, PA) was used as a substrate for glucose uptake in this study. Cells were cultured in 6-well cluster dishes, washed in Krebs-Ringer-HEPES (KRH) buffer (25 mM HEPES, pH 7.4, 118 mM NaCl, 4.8 mM KCl, 1.3 mM CaCl_2_, 1.2 mM KH_2_PO_4_, 1.3 mM MgSO_4_, 5 mM NaHCO_3_, 0.07% bovine serum albumin, and 5.5 mM glucose), and incubated for 20 min in KRH buffer. The cells were then incubated for another 10 min in KRH buffer containing 0.5 μCi of 2-deoxy-d-[^3^H]glucose. The reaction was terminated by placing the plates on ice and washing the cells in KRH buffer, after which they were lysed in 0.5 ml of 0.5N NaOH. Tracer activities were assessed using a liquid scintillation counter and the remaining volume of each sample was used to access the protein content per well by the Lowry method [30].

### Mitochondrial DNA sequencing

Mitochondrial DNA was amplified from total genomic DNA using REPLI-g mitochondria DNA (Qiagen, Valencia, CA). Mitochondrial sequencing was performed with a variant SEQr resequencing system (Applied Biosystems, Carlsbad, CA) and completed with an Applied Biosystems VariantSEQR Resequencing System and BigDye Terminator v3.1 Cycle Sequencing Kit; the samples were run on a 3100 Genetic Analyzer. Each PCR primer was tailed with M13 forward or reverse primer for subsequent sequencing reactions. Amplifications were performed as follows: 5 min at 96°C; 40 cycles of 30 s at 94°C, 45 s at 65°C, and 45 s at 72°C; and finally, 10 min at 72°C. The amplified products were separated by gel electrophoresis and then the DNA bands were eluted from the gel using a HiYield Gel/PCR DNA extraction kit (Real Biotech Corporation, Taipei, Taiwan). A VariantSEQr Resequencing System (Applied Biosystems) and ABI Prism Big Dye Terminator Cycle Sequencing Ready Reaction Kits were used for comparative sequencing following the manufacturer’s recommendations. All sequences were matched with the mitochondrial genome using NCBI-BLAST.

### Total RNA extraction and quantitative real-time reverse-transcription polymerase chain reaction (RT-PCR)

The total RNA from cells was extracted using TRIzol reagent (Invitrogen, USA). cDNA was synthesized by reverse-transcribing 3 μg of RNA at 42°C for 1 h in the presence of Avian Myeloblastosis Virus (AMV) reverse transcriptase, 100 nM oligo dT, 1 mM dNTP mixture, and RNase inhibitor. Real-time PCR was performed using a real-time SensiMix*Plus* SYBR kit (Quantance, London, UK) in a Rotor-Gene 3000 (Corbett Robotics, San Francisco, CA) as prescribed by the manufacturer’s instructions. The following primers for real-time PCR were used: 5′-AAGGCCAACCGCGAGAAGAT-3′ and 5′-CCAGAGGCGTACAGGGATAGCAC-3′ for human β-actin, and 5′-TTTTACCATGCCCCAGATTCA-3′ and 5′-AGTGCTTCCATCGGAAGGACT-3′ for human HIF-1α. The PCR cycling conditions were as follows: 15 min at 95°C; 40 cycles of 10 s at 95°C, and 15 s at 60°C; and 20 s at 72°C.

### Western blot analysis

Equal amounts of protein were analyzed in duplicate by sodium dodecyl sulfate polyacrylamide gel electrophoresis (SDS-PAGE). The following monoclonal antibodies were used: anti–phospho-Akt (Ser473, Cell signaling, Danvers, MA), anti–total Akt (Cell signaling), anti-phospho-mTOR (Ser2448, Cell signaling), anti-phospho-mTOR (Thr2446, Millipore, Bedford, MA), anti-total mTOR (Cell signaling), anti-HIF-1α (Cayman Chemical, Ann Arbor, MI), anti-VHL (Cell signaling), anti-phospho-AMPK (Thr172, Cell signaling), anti-total AMPK (Cell signaling) anti-phospho-P70S6K (Thr389, Cell signaling), anti-total P70S6K (Cell signaling), anti-phospho-4E-BP (Thr37/46, Cell signaling), anti-lactate dehydrogenase A (LDHA) (Cell signaling), anti-hexokinase-2 (HK-2, SantaCruz Biotechnology, Dallas, TX), anti-phospho-TSC2 (Ser1387) (Cell signaling), anti-total TSC2 (Cell signaling), and anti-β-actin (Bethyl, Montgomery, TX, US). Immunoreactive proteins were detected by horseradish peroxidase–conjugated secondary antibodies and enhanced using chemiluminescence (ECL) reagents (GE Healthcare Life Sciences, Piscataway, NJ). All immunoblots were performed with triplicate and visualized by LAS image analyzer (Fujifilm, Tokyo, Japan). The band density was quantified using MultiGauge (Fujifilm).

### Nuclear extracts and electrophoretic mobility shift assay

Nuclear lysates were prepared as previously described [31]. Briefly, cells were harvested, washed 3 times with PBS, resuspended in a hypotonic buffer (10 mM HEPES-KOH at pH 7.9, 1.5 mM MgCl_2_, 10 mM KCl, and 0.1% NP40), and incubated on ice for 10 min. Nuclei were precipitated by centrifugation at 12,000 ×*g* for 10 min at 4°C. After washing once with the hypotonic buffer, the nuclei were lysed in a lysis buffer (50 mM Tris-HCl at pH 8.0, 150 mM NaCl, and 1% Triton X-100), incubated on ice for 30 min, and pre-cleared by centrifugation at 20,000 ×*g* for 15 min at 4°C. For electrophoretic mobility shift assay (EMSA) experiments, HIF-1α probes were labeled, by combining 250 ng of the annealed oligonucleotide; 2 μl of 10× T4 kinase buffer (Takara Bio, Otsu, Japan); 1 μl each of a 5 mM stock of dATP, dTTP, dCTP, and dGTP; 8 μl of water; 1 μl of T4 polynucleotide kinase (Takara Bio); and 5 μCi of [^32^P]dCTP (GE Healthcare Bio-Sciences) at 37°C for 1 h. Ten micrograms of nuclear protein extract were incubated in 20 μl of a solution of 10 mM HEPES at pH 7.9, 80 mM NaCl, 10% glycerol, 1 mM DTT, 1 mM EDTA, 100 μg/ml poly (deoxyinosinic-deoxycytidylic acid), and radiolabeled double-stranded oligonucleotide (5′-GAGCGGACGTGCTGGCGTGGCACGTCCTCTC-3′, 5′-GAGAGGACGTGCCACGCCAGCACGTCCGCTC-3′) containing the HRE elements that are present in the human heme oxygenase-1 promoter. Binding between oligonucleotides and HRE was induced by incubating the samples at 37°C for 20 min. Final binding complexes were loaded onto 6% polyacrylamide gels. The gels were then dried and separated at 150 V for 2.5 h. Competition experiments were performed by adding 50-fold and 100-fold excess of unlabeled oligonucleotides.

### Cell cytotoxicity assay

Cells were plated at 3 × 10^3^ cells/well in 96-well plates and grown for 24 h in standard culture media. The cells were then treated with 10 μM of tamoxifen citrate for 24 h. After treatment, the medium was discarded and the proliferation of each cell line was measured using an XTT assay kit (Roche Diagnostics GmbH, Mannheim, Germany) according to the manufacturer’s instruction. The absorbance of the converted dye was measured at a wavelength of 470 nm.

To confirm the cell proliferation affected by glycolysis inhibitor or glycolytic enzyme, cells were also plated at 3 × 10^3^ per well in 96-well plates and grown for 24 h with standard culture media. The cells were treated with 3-Bromopyruvate (3-BrPA) in a range of 50 to 200 μM for 24 h. A 0.1% v/v DMSO solution in standard culture media was used as the vehicle control. After treatment, the proliferation of each cell line was measured using an XTT assay kit. Another set of plated cells was transfected with HK-2 siRNA (sense 5’-CCUGGGUGAGAUUGUCCGUAA-3’ and antisense 5’-UUACGGACAAUCUCACCCAGG-3’) or control siRNA with 20 nM for 24 h and the cell proliferation was measured after 48 h transfection by using XTT assay kit.

### Statistical analysis

Statistical significance was examined using the Student’s *t* test. A two-sample *t* test was used for comparisons between two groups. Values were reported as means ± SD. *P* < 0.05 was considered to indicate statistical significance.

## Results

### Increased glycolytic rate in tamoxifen-resistant breast cancer cells

**Fig 1:**
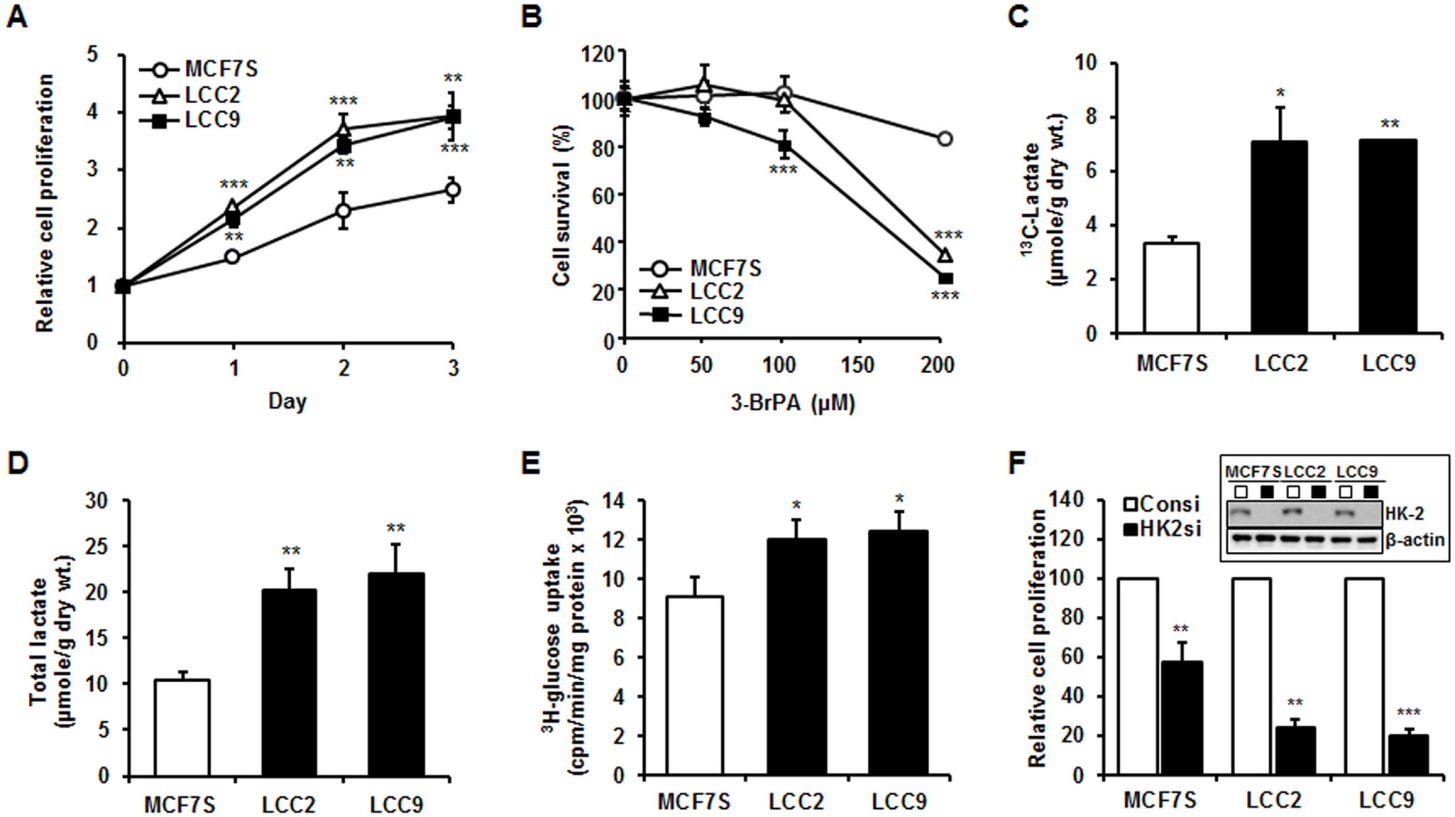
Lactate production and glucose consumption were elevated in LCC2 and LCC9 cells. (A) Cell proliferation rate of MCF7S, LCC2 and LCC9 cells was measured by XTT assay in a time-dependent manner. The data represent the mean ± SD of representative results for 3 independent assays. (B) Viability of MCF7s, LCC2 and LCC9 cells was measured by XTT assay 24 h after the treatment various concentration of 3-BrPA. The data represent the mean ± SD of representative results for 3 independent assays. (C) The amount of ^13^C-labeled lactate was determined in each cell line by ^1^H NMR quantification. ^13^C-labeled lactate derived from glycolysis was traced and quantified for each cell line. Spectra were normalized to the cell dry weight and an NMR scaling factor. (D) The amount of total lactate in the cell extracts was quantified through GC-MS analysis. The vertical axis represents the amount of total lactate (μM) per dry weight of protein (g). (E) ^3^H-glucose was used to treat each cell line for 2 h. Glucose uptake was expressed as counts per minute per milligram of protein (cpm/min/mg protein). (F) Inhibition of hexokinase-2 (HK-2) repressed cell proliferation, especially in LCC2 and LCC9, compared with MCF7S. Control siRNA (Consi) and HK-2 siRNA (HK2si) were transfected into each cell line with 20 nM for 24 h and cell proliferation was measured after 48 h transfection. The data represent the mean ± SD of 3 independent experiments that were performed in triplicate. *, *P* < 0.05; **, *P* < 0.01; ***, *P* < 0.001.

Tamoxifen-resistant cells grew more rapidly than non-resistant cells, implying that the increased glycolysis of tamoxifen-resistant cells is at least due, in part, to faster growth of these cells (Fig 1A). To determine the sensitivity toward tamoxifen and a role for a glycolysis inhibitor on the drug sensitivity, tamoxifen citrate or 3-BrPA was treated to various cell lines and cell viability was determined using XTT assay. The growth of MCF7S cells was inhibited by tamoxifen citrate, but not those of LCC2 and LCC9 cells (S1 Fig). In addition, LCC2 and LCC9 cells were more sensitive to treatment of 3-BrPA than MCF7S in a 3-BrPA concentration-dependent manner (Fig 1B). Furthermore, treatment of 3-BrPA induced cell cycle arrest in LCC2 and LCC9 cells: significant increases (by 7.1% and 6.9%) were observed in the percentages of cells at G_1_ phase in LCC2 and LCC9 cells, respectively (S2 Fig). This result suggests that glycolysis inhibitor, 3-BrPA, can sensitize tamoxifen-resistant cells. The levels of lactate produced via the aerobic glycolysis of ^13^C_6_-glucose labeled glucose were monitored using 1D and 2D ^1^H NMR methods to assess if the tamoxifen-resistant breast cancer cells had a higher rate of aerobic glycolysis. Approximately 2.5-fold increases in ^13^C_3_-lactate were observed in the LCC2 and LCC9 cells, but not in the MCF7s cells (Fig 1C and S3 Fig), which implies a higher glycolytic flux in the tamoxifen-resistant cells than in the non-resistant control cell line.

For absolute quantification of glycolysis-related metabolites the amount of total lactate produced in the cells was resolved and quantified by GC-MS. As observed in previous experiments, total lactate was increased in the LCC2 and LCC9 cells compared to the MCF7S cells (Fig 1D). In addition, the concentration of incorporated [^3^H]-labeled glucose was slightly higher in the LCC2 and LCC9 cells compared to the MCF7S cells (Fig 1E). These data demonstrate that the tamoxifen-resistant MCF7 sub-lines LCC2 and LCC9 displayed increased lactate production and higher glucose uptake compared to the control cells and that they undergo aerobic glycolysis at higher rates.

In order to further demonstrate the association of increased glycolysis with tamoxifen resistance, expression of the gene critical for the glycolytic pathway was downregulated using the siRNA approach. The target gene of the siRNA approach is the glycolytic enzyme hexokinase 2 (HK-2), which is predominantly overexpressed in malignant tumors and crucial for the Warburg effect. Knock-down of HK-2 exacerbated cell viability of three cell lines, in which cell proliferation was dramatically reduced in tamoxifen-resistant cells by 70~80% whereas only 40% inhibition of cell viability was observed in MCF7S cells (Fig 1F). Our results showed that glycolysis is crucial for the survival of tamoxifen-resistant cells.

### Mitochondrial malfunction in tamoxifen-resistant breast cancer cells as a mechanism of elevated aerobic glycolysis

High rates of glycolysis in cancer cells have been suggested to be as a result of impairment in mitochondrial respiration [32]. To investigate whether the Warburg effect seen in the LCC2 and LCC9 cell lines was attributed to the lack of normal oxidative phosphorylation via mitochondrial malfunction, the formation of the endogenous reactive oxygen species (ROS) was compared in a time-dependent manner in these three cell lines using fluorescent probes. Intracellular basal ROS production did not significantly differ among the parent and tamoxifen-resistant cells (S4A and S4B Fig). To further confirm endogenous ROS levels, the concentrations of intracellular reduced glutathione (GSH) and oxidized glutathione (GSSG) were measured. The GSH:GSSG ratios were statistically different, this difference is not biologically meaningful (S4C Fig). Under oxidative stress, the formation of GSSG in cells is increased, and thus, the ratio of GSH to GSSG is reduced. Our results suggest that tamoxifen-resistant cells generally were not exposed to unusual oxidative stress compared to the parent cell line. Moreover, the oxygen consumption rates (OCRs) were also similar in each cell line (S4D Fig), indicating that mitochondrial function was not an important factor in the acquisition of tamoxifen resistance or increased aerobic glycolysis.

To confirm the potential defects in mitochondria that were present in these cell lines in addition to ROS production by mitochondria, full mitochondrial genome sequencing was performed using the mitochondrial DNA prepared from the nuclear extracts from each cell line. A change in only a single amino acid for the cytochrome B (CYTB) gene was identified in both the LCC2 and LCC9 cell lines, but not in MCF7S (Table 1). The effects of this single mutation on the mitochondrial function, in terms of oxidative phosphorylation and the TCA cycle, are still unknown. Taken together, these results suggest that mitochondrial malfunction does not play a significant role in increased aerobic glycolysis in tamoxifen-resistant cells.

**Table 1:** Somatic mtDNA mutations in MCF7S, LCC 2, and LCC9 cells.

mtDNA(AC_000021.2)			MCF7S			LCC2			LCC9		
Locus	Gene*	Reference base	Base change	Codon change	Amino acid change	Base change	Codon change	Amino acid change	Base change	Codon change	Amino acid change
195	D-L	C	>T	NPCL	-	>T	NPCL	-	>T	NPCL	-
315	D-L	C	Insert C	NPCL	-	Insert C	NPCL	-	Insert C	NPCL	-
752	12S rRNA	A	>G	NPCL	-	>G	NPCL	-	>G	NPCL	-
1440	12S rRNA	A	>G	NPCL	-	>G	NPCL	-	>G	NPCL	-
4769	ND2	A	>G	ATA>ATG	-	>G	ATA>ATG	-	>G	ATA>ATG	-
5896–5900	NPCL	CCCCC	Insert CC	NPCL	-	Insert CC	NPCL	-	Insert CC	NPCL	-
6777	COX1	T	>C	CAT>CAC	-	>C	CAT>CAC	-	>C	CAT>CAC	-
8860	ATPase	A	>G	ACA>GCA	T112A	>G	ACA>GCA	T112A	>G	ACA>GCA	T112A
9967	COX3	G	>A	GTC>ATC	V254 I	>A	GTC>ATC	V254 I	>A	GTC>ATC	V254 I
13261	ND5	T	>C	AGT>AGC	-	>C	AGT>AGC	-	>C	AGT>AGC	-
15326	CYTB	A	>G	GCA>GCG	-	>G	GCA>GCG	-	>G	GCA>GCG	-
15381	CYTB	A	>G	ACC>GCC	T212A	>G	ACC>GCC	T212A	>G	ACC>GCC	T212A
15839	CYTB	C	>C	-	-	>A	ATC > ATA	I364M	>A	ATC > ATA	I364M
16149	D-L	C	>T	NPCL	-	>T	NPCL	-	>T	NPCL	-
16519	D-L	C	>T	NPCL	-	>T	NPCL	-	>T	NPCL	-

*D-L, D-loop; 12S, 12S ribosomal RNA; ND2, 5 NADH dehydrogenase subunit 2, 5; ATPase, ATP synthase F0 subunit 8; COX3, cytochrome C oxidase subunit III; CYTB, cytochrome B; and NPCL, non-protein coding locus

### Increased aerobic glycolysis by upregulated HIF-1α through Akt/mTOR activity

**Fig 2:**
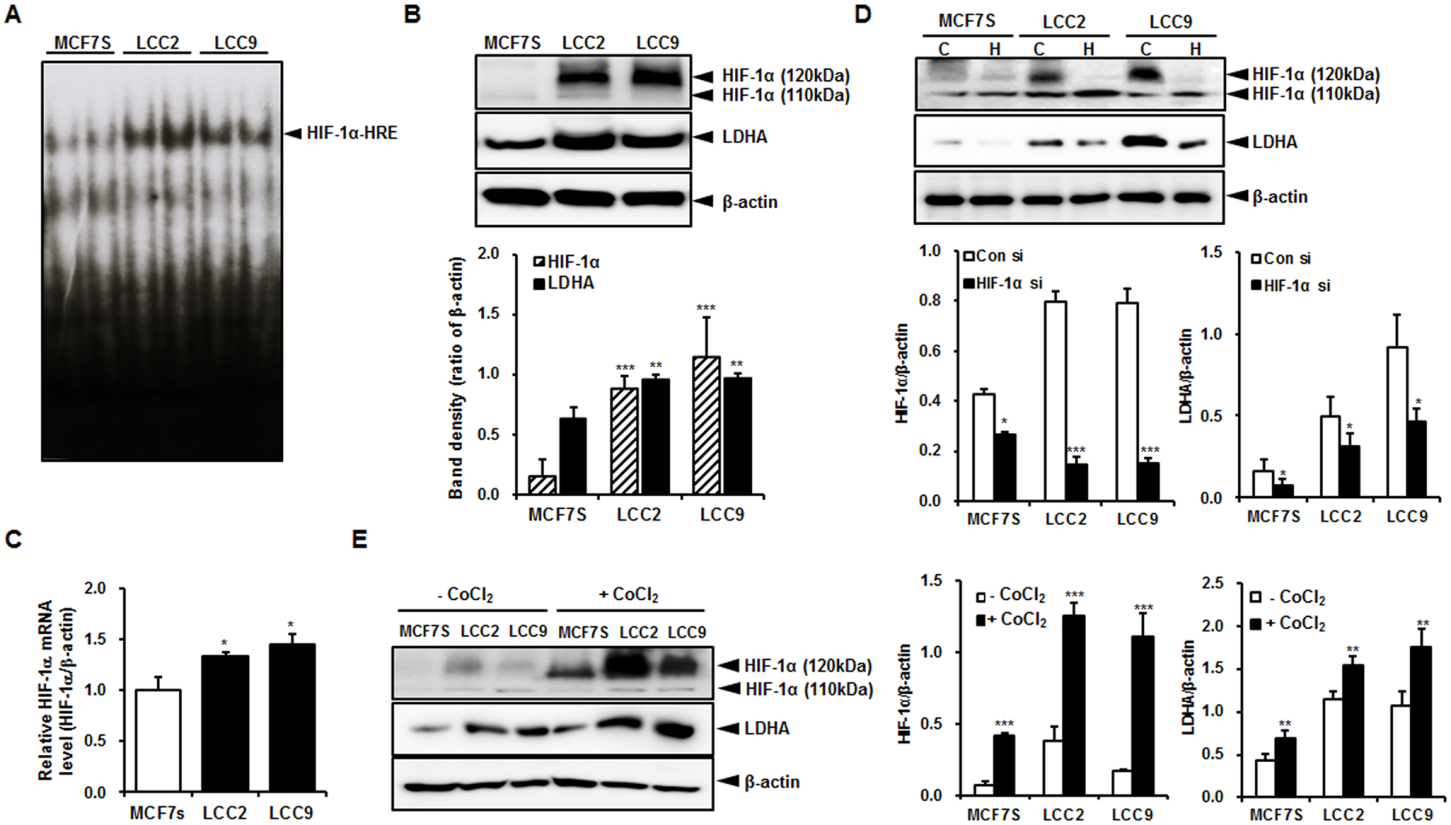
HIF-1α is upregulated in LCC2 and LCC9 cells. (A) HIF-1α activity in MCF7S, LCC2 and LCC9 cells was measured by EMSA. (B) Endogenous HIF-1α and LDHA protein levels were measured by western blotting. β-Actin was used as a loading control. (C) The mRNA level of HIF-1α was measured by quantitative RT-PCR using the β-actin gene as an internal control. (D) LDHA was downregulated in MCF7S, LCC2, and LCC9 cells transfected with HIF-1α siRNA. siRNA (20nM) was treated in three cell lines for 48 h. C, control siRNA; H, HIF-1α siRNA (E) HIF-1α was overexpressed by treatment of CoCl_2_ (50 μM, 24 h) in each cell line. Elevation of HIF-1α induced increase in LDHA expression levels. The data shown are representative results of 3 independent experiments. Band density of each immunoblot was quantified using MultiGauge. *, *P* < 0.05; **, *P* < 0.01; ***, *P* < 0.001.

To find any key molecules related to increased aerobic glycolysis in association with tamoxifen resistance, the HIF-1α activity was measured by EMSA. LCC2 and LCC9 cells showed markedly higher binding activity to HRE than that of MCF7S cells (Fig 2A). In addition, the amounts of protein under normal culture conditions were clearly increased in the LCC2 and LCC9 cells, but were not visible in the MCF7S cells (Fig 2B), while transcript level of HIF-1α were slightly increased in LCC2 and LCC9 cells (Fig 2C). Hyperactivation of HIF-1α induced LDHA upregulation in LCC2 and LCC9 cells (Fig 2B). This result was confirmed by inducing the inhibition and activation of HIF-1α by siRNA and the CoCl_2_-induced hypoxia condition (Fig 2D and 2E). Therefore, these results suggest that HIF-1α activation may be responsible for lactate accumulation, by promoting LDHA and the acquisition of tamoxifen resistance, in LCC2 and LCC9 cells.

**Fig 3:**
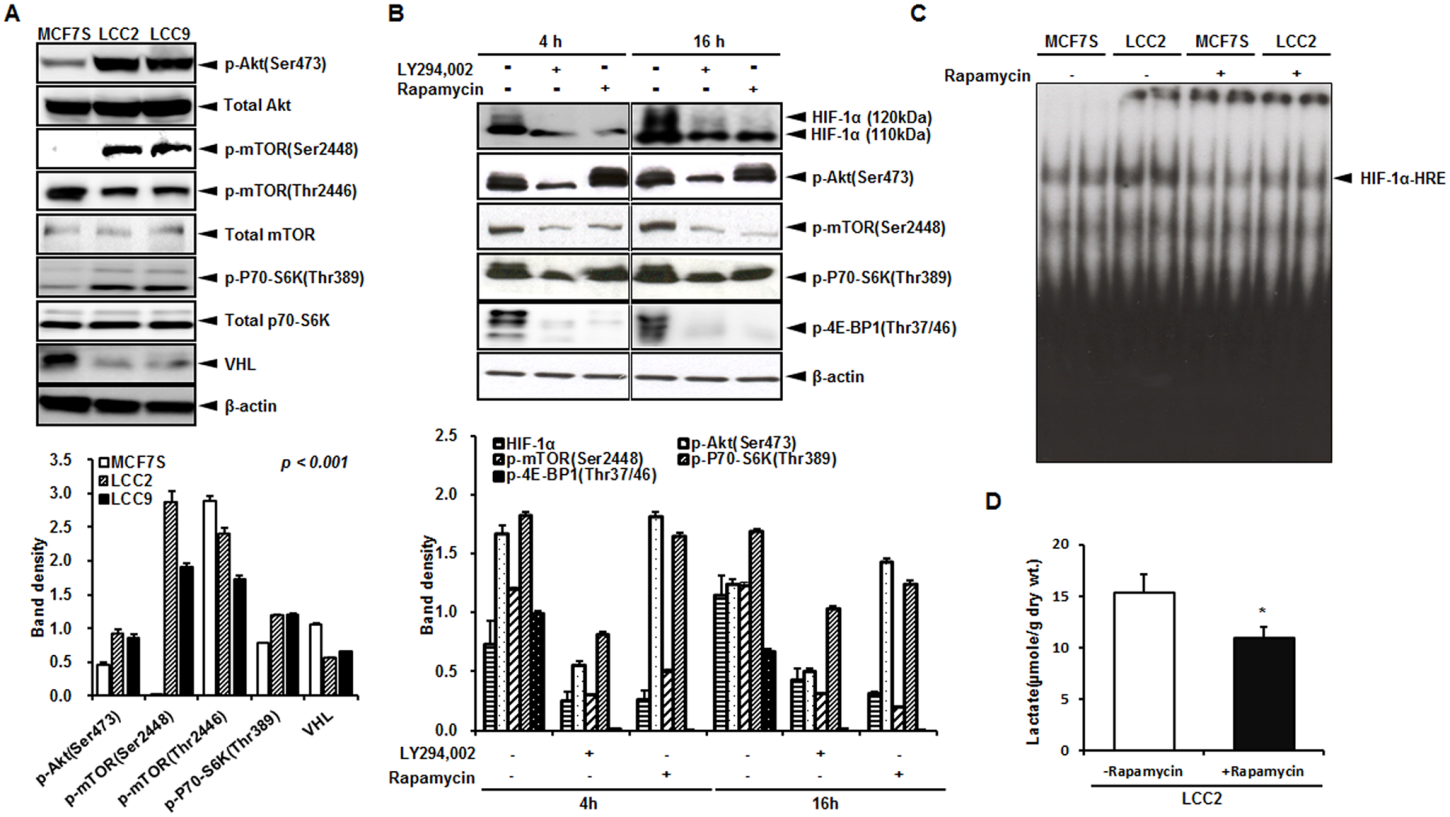
Increased Akt/mTOR signaling induces HIF-1α activity and aerobic glycolysis. (A) VHL protein levels, as well as the activity of Akt/mTOR and its downstream targets measured by western blotting using β-actin as a loading control. (B) After treatment with specific inhibitors of Akt (LY294,002, 40 μM) and mTOR (rapamycin, 30 nM) for 4 and 16 h, respectively, HIF-1α and Akt/mTOR protein expressions were measured by western blotting in LCC2 cells. β-Actin was used as a loading control. Band density of each immunoblot with triplicate was quantified using MultiGauge. (C) Rapamycin at 30 nM was used to treat LCC2 cells for 16 h. HIF-1α activity was measured using EMSA in LCC2 cells. The data represent results of 3 independent experiments. (D) Lactate accumulation was measured by ^1^H NMR quantification after treatment of rapamycin (30 nM, 16 h). *, *P* < 0.05.

To investigate whether the HIF-1α protein was upregulated by enhancing its stability through VHL inactivation or the translational efficiency of mRNA through the mTOR pathway, VHL, Akt, and mTOR activities were measured by western blotting. Although VHL protein levels were decreased in LCC2 and LCC9 compared to the control, Akt/mTOR and its downstream target proteins were significantly hyperphosphorylated in the LCC2 and LCC9 cells (Fig 3A). This data indicates a significantly higher translation of HIF-1α mRNA by Akt/mTOR activity as well as VHL-dependent ubiquitination in the LCC2 and LCC9 cells even under oxygen-rich conditions.

To verify if this higher activity of Akt was specific to the upregulation of mTOR activity, thereby leading to increased translation of HIF-1α mRNA, LCC2 cells were treated with inhibitors for either phosphoinositide 3-kinase (PI3K) or mTOR in a time-dependent manner. Both inhibitors effectively reduced HIF-1α activity (Fig 3B and 3C). Collectively, our data indicate that increased HIF-1α activity in LCC2 and LCC9 cells may be regulated by the Akt/mTOR pathway. Blocking mTOR reduced lactate accumulation in the LCC2 cells (Fig 3D), indicating that increased aerobic glycolysis in the LCC2 and LCC9 cells was mediated through HIF-1α that was upregulated by the Akt/mTOR pathway.

### Restoration of the Akt/mTOR/HIF-1α signaling by inhibiting glycolytic enzyme in tamoxifen-resistant breast cancer cells

**Fig 4:**
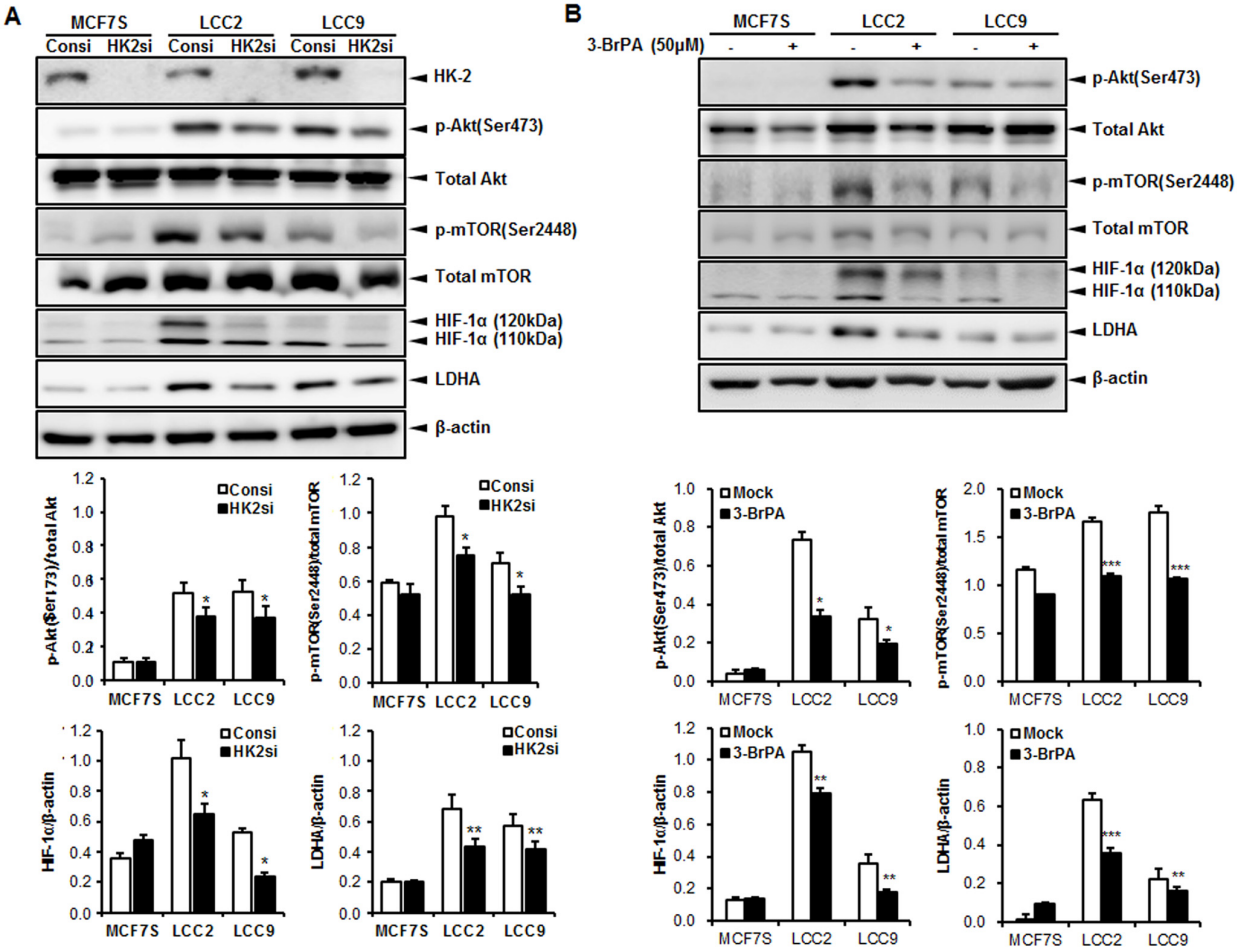
Akt/mTOR/HIF-1α axis was specifically suppressed by knockdown of hexokinase-2 (HK-2) glycolytic enzyme in LCC2 and LCC9 cells. (A) Cells were transfected with control siRNA (Consi) or HK-2 siRNA (HK2si) at 20 nM for 48 h followed by treatment of 4-OHT (10 μM, 48 h). (B) Cells were treated with 3-BrPA (50 μM) for 4 h and followed by treatment of 4-OHT (10 μM, 48 h). Western blotting was performed for the lysates prepared from treated cells. β-Actin was served as a loading control. The data shown are representative results for at least 3 independent experiments. Band density was quantified using MultiGauge. *, *P* < 0.01; **, *P* < 0.001 vs. Consi transfected cells.

To prove if Akt/mTOR/HIF-1α axis is involved in elevated aerobic glycolysis in tamoxifen-resistant breast cancer cells, their phosphorylation status or expression levels were investigated by western blot analysis after knockdown of HK-2 or 3-BrPA treatment followed by 4-OHT treatment in three cell lines. Interestingly, both silencing of HK-2 by siRNA approach and treatment of 3-BrPA significantly reduced Akt/mTOR/HIF-1α axis in both LCC2 and LCC9 cells since the levels of phosphorylated forms of Akt and mTOR as well as the protein level of HIF-1α were decreased in siRNA or 3-BrPA-treated samples (Fig 4A and 4B). It is notable that the activated forms of both Akt and mTOR and the basal levels of HIF-1α were observed in an insignificant amount in MCF7S regardless of HK-2 inhibition. Decreased signaling activity of Akt/mTOR axis led to downregulation of LDHA protein level in both LCC2 and LCC9 cell lines. Therefore, in line with Fig 1F, this data suggests that control of aerobic glycolysis can overcome the tamoxifen resistance of breast cancer cells by regulating the key signaling pathway of Akt/mTOR/HIF-1α axis.

### Inhibitory effect on aerobic glycolysis by AMPK activation

**Fig 5:**
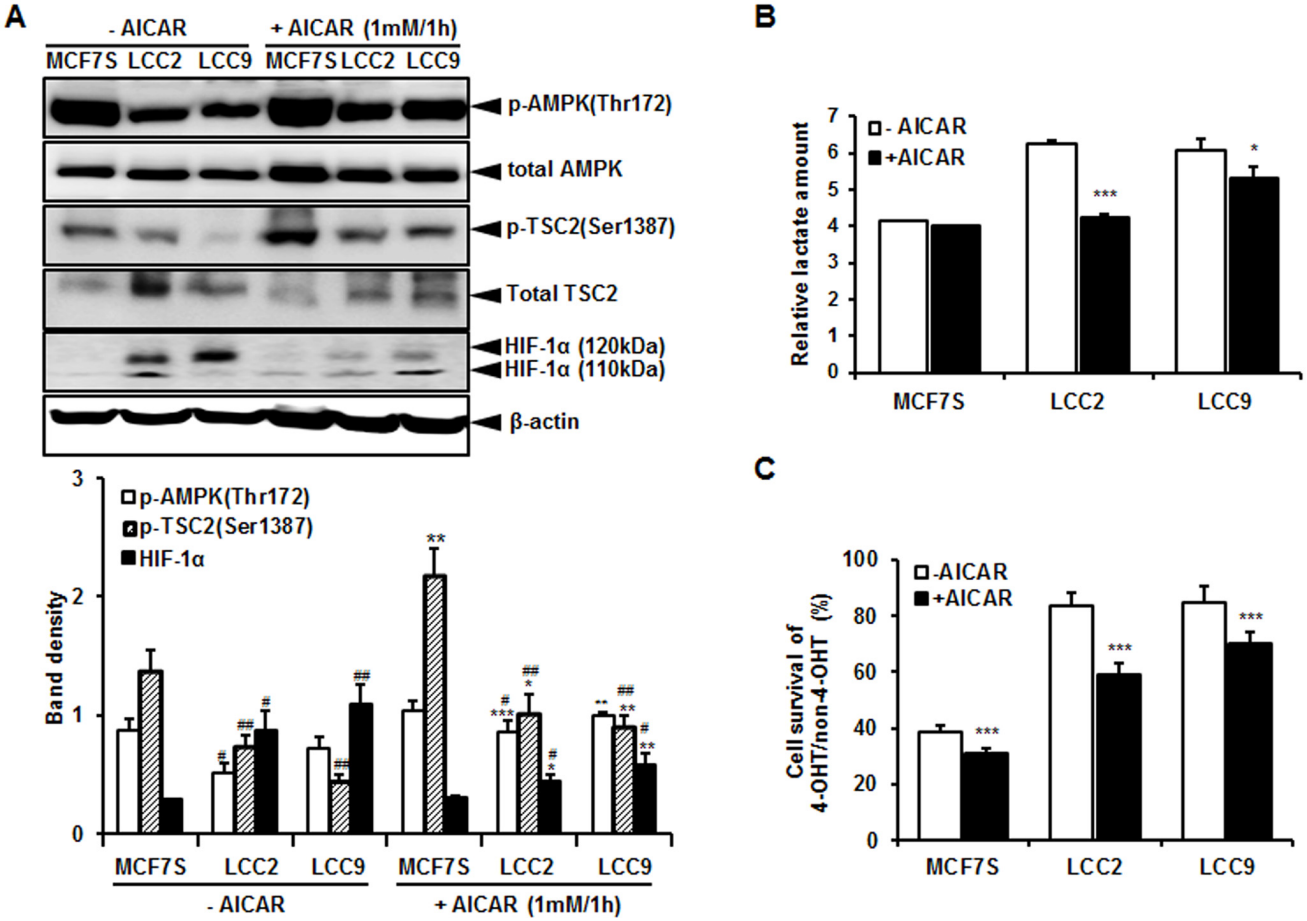
AMPK activation was decreased in LCC2 and LCC9 cells compared to MCF7S cells. (A) Cells were treated with 1 mM AICAR for 60 min. p-AMPK (Thr172), p-TSC2 (Ser1387), and HIF-1α was detected by western blotting. β-Actin was served as a loading control. The data shown are representative results for at least 3 independent western blot analyses. Band density was quantified using MultiGauge. *, *P* < 0.05; **, *P* < 0.01; ***, *P* < 0.001 vs. no treatment of AICAR and ^#^, *P* < 0.05; ^##^, *P* < 0.01; ^###^, *P* < 0.001 vs. MCF7S control cells. (B) AICAR-treated cells were assayed for lactate production. (C) Cells treated with AICAR were treated with 4-OHT (5 μM) for 3 days and cell survival of MCF7S, LCC2, and LCC9 cells was measured by XTT assay. The data represent the mean ± SD of representative results for 3 independent lactate assays. *, *P* < 0.05; ***, *P* < 0.001.

To determine whether AMPK, which is an upstream regulator of mTOR, increases HIF-1α and aerobic glycolysis in LCC2 and LCC9 cells, AMPK activity was evaluated by western blotting analysis. As a result, basal levels of p-AMPK were higher in the MCF7S cells than in the LCC2 and LCC9 cells. When treated with AICAR, which is an activator of AMPK, the overall AMPK activity increased more in the MCF7S cells, than in the LCC2 and LCC9 cells. Also, AICAR treatment increased p-TSC2 (Ser1387), which is direct target of AMPK. HIF-1α levels in the LCC2 and LCC9 cells were also reduced upon AMPK activation but to a lesser extent in the MCF7S cells (Fig 5A). This altered signaling by AMPK hyperactivation caused a reduction of lactate accumulation, especially in the LCC2 and LCC9 cells (Fig 5B). Furthermore, as shown in Fig 5C, AICAR reduced cell survival in response to tamoxifen treatment in LCC2 and LCC9 cell lines. Interestingly, the cell survival rate was more reduced in tamoxifen-resistant cells compared to that in MCF7S cells. These data indicate that AMPK may also affect the HIF-1α activity and HIF-1α-associated aerobic glycolysis.

## Discussion

In this study, we demonstrate that tamoxifen-resistant breast cancer cells express higher levels of HIF-1α than parental breast cancer cells and it is closely associated with elevated aerobic glycolysis. Several factors, including hypoxia and oncogenic events, have been linked to the stabilization of HIF-1 in the presence of oxygen leading to transactivation of genes that code for glycolytic enzymes and enzymes that convert glucose into lactate [11]. Since the induction in HIF-1 activity seems to play a critical role in cancer metabolism, the inhibition of HIF-1 has been implicated in blocking tumor growth and progression in animal model studies [33]. Increased HIF-1α activity stimulates the expression of the key enzymes that carry out glucose metabolism, including LDHA, pyruvate dehydrogenase kinase-3, and glucose transporter 1. Actually, we confirmed that increased protein expression of HIF-1α was concomitant with the upregulation of LDHA in tamoxifen-resistant cells. LDHA regenerates the nicotinamide adenine dinucleotide (NAD^+^) which is required to maintain glycolysis by converting pyruvate to lactate and it is one of the main isoforms of LDH found in breast tissue [34]. It has been reported that the inhibition of LDHA could reverse taxol resistance in breast cancer cells, which implies the role of glycolytic enzymes and the Warburg effect in chemoresistance [35]. Taken together, our data indicate that HIF-1α hyperactivity was associated with increased aerobic glycolysis in our tamoxifen-resistant model cell lines.

Warburg effect has been attributed to unique mechanism by which cancer cells produce ATP for survival. In addition, studies have shown that changes in glucose metabolism and ATP production play a role in chemoresistance [12,36,37]. An MCF7 sub-line that was resistant to adriamycin showed a three-fold faster rate of lactate production compared to drug-sensitive MCF7 cells, and accordingly, glucose depletion caused a dramatic decrease in ATP production in the resistant cells [12]. These results indicate that drug-resistant cells are likely to develop a greater dependence on glucose metabolism due to a high demand for ATP, which implies that energy-consuming mechanisms prevail for drug-resistant cells to survive. Indeed, in this study, either treatment with 3-BrPA or inhibition of key glycolytic enzyme resulted in dramatic decrease in cell survival among the tamoxifen-resistant cells, but rarely in the tamoxifen-sensitive cells. This demonstrates that tamoxifen-resistant cells strongly depend on aerobic glycolysis for their energy supply and that the inhibition of glycolysis may be a rational strategy to selectively kill tamoxifen-resistant cells. Thus, targeting dysregulated glycolysis was suggested as a novel strategy to sensitize cancer cells to chemotherapy drug resistance [35,36,38]. The recent research on the regulation of glucose metabolism and breast cancer treatment suggested that alterations in bioenergetics play a role in resistance to trastuzumab and glycolysis inhibition in combination with trastuzumab induced more efficient inhibition of glycolysis in ErbB2-positive breast cancer cells [38]. In addition, another group also suggested that modulation of glycolysis targeting the upstream regulator of Akt ubiquitination resensitizes ErbB2-positive breast tumors to trastuzumab [39]. These data clearly imply the importance of glycolysis as a therapeutic target in treatment of breast cancer.

Several oncogenes have been identified as the main players associated with the Warburg effect via the activation of HIF-1α. For example, the *AKT* oncogene is associated with increased glycolytic flux without affecting mitochondrial oxidative phosphorylation thereby contributing to the Warburg effect [40,41]. Akt also regulates the acute expression of glycolytic enzymes, which leads to an increase in aerobic glycolysis [41]. The signaling pathway composed of PI3K, Akt, and mTOR has been shown to be associated with the upregulation of HIF-1α whose activation also contributes to the Warburg effect [11]. It has been reported that high Akt activity is linked with a poor prognosis as well as endocrine resistance [42]. Therefore, Akt hyperactivation may protect tumor cells from energy deprivation by constitutive ATP production and drug-induced apoptosis by mTOR activation, which is highly activated in tumor cells, and contribute to cell survival and growth [23]. Several studies have shown that targeting Akt or mTOR restores the response to tamoxifen or trastuzumab in breast cancer cells, which implies causative effects of hyperactivation of Akt/mTOR during the development of drug resistance [42–47]. Despite many studies on the abnormal glucose metabolism in terms of chemoresistance in various types of cancers and the hyperactivation of Akt/mTOR in tamoxifen-resistant breast cancer, there is no report to date regarding the roles of the Warburg effect in tamoxifen resistance and signaling molecules or pathways associated with the switch in energy dependence in tamoxifen-resistant cells. In our study, roles of Akt and mTOR in relation to glucose metabolism were investigated in tamoxifen-resistant cells. Inhibition of mTOR and/or PI3K by chemical inhibitors led to downregulation of HIF-1α protein and suppression of interaction between HIF-1α and HRE, implying that activity of HIF-1α is regulated via an Akt/mTOR-mediated pathway in tamoxifen-resistant cells. Finally, inhibition of the Akt/mTOR pathway resulted in less accumulation of lactate in the LCC2 cells and glycolysis inhibition induced repression of Akt/mTOR/HIF-1α axis in tamoxifen-resistant breast cancer cell-specific manner. These data strongly indicates that aerobic glycolysis in tamoxifen-resistant cells is controlled through Akt/mTOR/HIF-1α pathways. In addition, the activation of HIF-1α is mediated via a VHL-independent pathway and may result from the activation of oncogene Akt/mTOR signaling pathways, thereby leading to the Warburg effect in LCC2 and LCC9 cells. Our study also suggests that targeting glycolysis may be an effective strategy to overcome tamoxifen resistance through a Akt/mTOR/HIF-1α pathway.

It is known that the nutrient sensing system AMPK pathway is a major regulator of mTOR activation in addition to the PI3K/Akt pathway. Originally, activation of AMPK via a low AMP/ATP ratio inhibits protein synthesis and the progression of cell cycle through mTOR/P70S6K signaling [22,48]. In this study, it was demonstrated that AICAR-stimulated activation of AMPK as well as the basal levels of activated AMPK was decreased more in the LCC2 and LCC9 cells compared to MCF7S cells. Constitutively activated Akt signaling and decreased AMPK activity may play crucial roles in the hyperactivation of HIF-1α protein, via mTOR signaling in LCC2 and LCC9 cells. This suggests that the AMPK pathway may be an alternative target for overcoming tamoxifen resistance. AMPK also regulates the activation of p53 tumor suppressor, which is important to G1 cell cycle arrest following glucose depletion [49]. It was reported that the lack of p53 is involved in HIF-1α stabilization [50] and the upregulation of glycolytic enzymes [51]. These observations suggest that aerobic glycolysis governs tumor cells via both Akt/mTOR/HIF-1α and AMPK pathways and that tumor cells may escape from apoptosis through mTOR activation.

## Conclusion

Our study reports herein that not only tamoxifen-resistant breast cancer cells display altered glycolysis compared to tamoxifen-sensitive cells but also Akt/mTOR and AMPK signaling molecules are attributed to HIF-1α-regulated glucose metabolism in these cells. Activated mTOR induces cap-dependent translation of HIF-1α, which results in aerobic glycolysis to produce ATP. Moreover, AMPK activation induced by AMPK activator negatively regulates the glycolysis-dependent metabolism in tamoxifen-resistant breast cancer cells by inhibiting mTOR/HIF-1α signaling.

The most importantly, this is the first report to demonstrate that alterations in glucose metabolism are revealed in tamoxifen-resistant breast cancer cells and that the HIF-1α pathway and Akt/mTOR oncogenic signaling molecules are associated with the Warburg effect in these cells. Modulation of glycolysis pathway via knockdown of the specific genes or chemical inhibitors can restore cellular sensitivity toward antiestrogen in drug-resistance cells. Our results provide insight into a rationale for devising more efficient endocrine regimens to overcome tamoxifen resistance by targeting aerobic glycolysis pathways.

## Supporting Information

S1 Fig LCC2 and LCC9 cells were resistant to tamoxifen even with high concentration of 10 μM. Cells were treated with tamoxifen citrate in a range of 1 to 10 μM for 24 h and cell viability was measured using SRB assay. *P* < 0.05.
(TIF)

S2 Fig Cell cycle analysis by flow cytometry after treatment of 3-BrPA into MCF7S, LCC2 and LCC9 cells. Cells were treated with 50 μM 3-BrPA 24 h post seeding. At 24 h post-treatment cells were harvested, stained with PI and analyzed for cell cycle distribution by flow cytometry using FlowJo software. Fluorescence histograms showing cell cycle distribution (G1, S, and G2/M phases) of each cell line and 3-BrPA treated cells.
(TIF)

S3 Fig Lactate ^1^H NMR spectra in MCF7s and LCC2 cell lysates (A) and medium (B). (TIF)

S4 Fig Endogenous ROS formation was not different between tamoxifen-sensitive and resistant breast cancer cells. (A–B) Harvested cells were treated with DCFH-DA (50 μg/ml) or DHE (10 μg/ml) for the measurement of intracellular hydrogen peroxide and superoxide levels, respectively. The detection was performed by time-kinetics over 1 h at 10 min intervals. DCFH-DA- or DHE-treated 1× PBS was used as a negative control. (C) The concentrations of intracellular GSH and GSSG were measured and the GSH:GSSG ratio was calculated in MCF7S, LCC2, and LCC9 cells. *, *P* < 0.01. (D) Oxygen consumption rate was measured in three cell lines. The data are the mean ± SD of 3 independent experiments.
(TIF)

S1 File Materials and methods. (PDF)
