# Supplementary figures and images for: Inhibition of Aerobic Glycolysis Represses Akt/mTOR/HIF-1α Axis and Restores Tamoxifen Sensitivity in Antiestrogen-Resistant Breast Cancer Cells
Source: PLoS One. 2015 Jul 9;10(7):e0132285. doi: 10.1371/journal.pone.0132285 (PMC4497721; doi:10.1371/journal.pone.0132285)

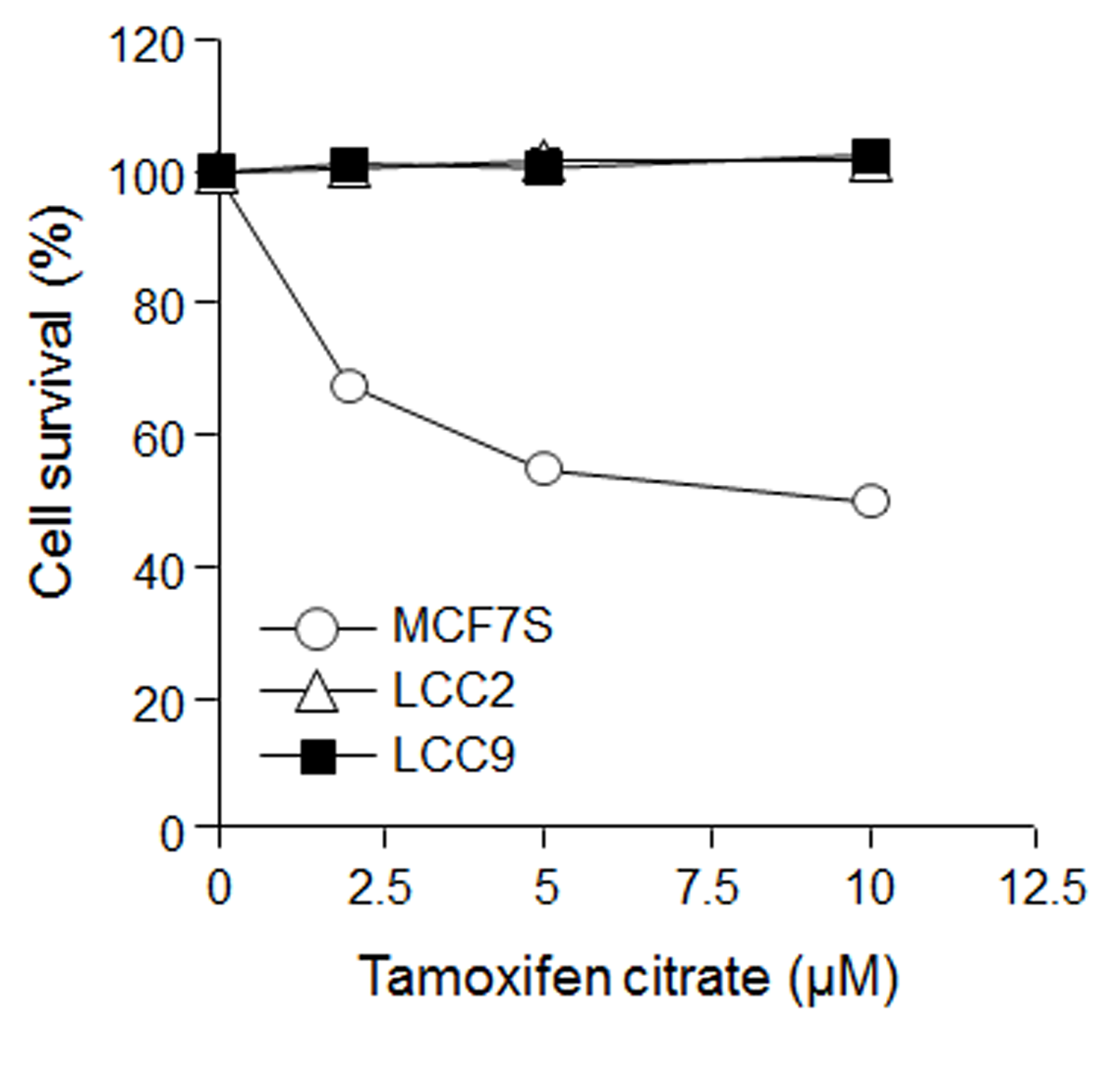

Supplement: S1 Fig — Cells were treated with tamoxifen citrate in a range of 1 to 10 μM for 24 h and cell viability was measured using SRB assay. P < 0.05. (TIF) [file pone.0132285.s001.tif]

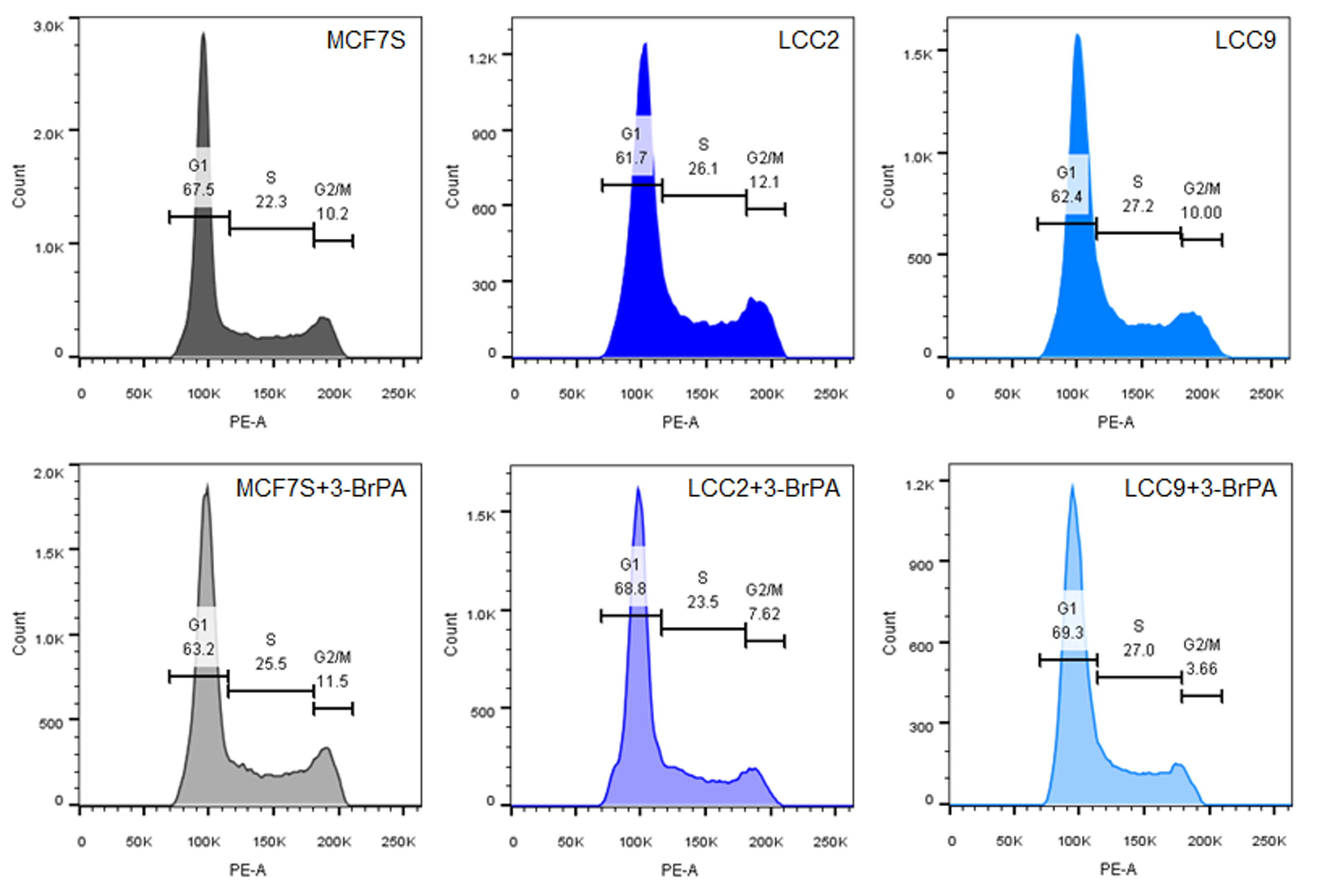

Supplement: S2 Fig — Cells were treated with 50 μM 3-BrPA 24 h post seeding. At 24 h post-treatment cells were harvested, stained with PI and analyzed for cell cycle distribution by flow cytometry using FlowJo software. Fluorescence histograms showing cell cycle distribution (G1, S, and G2/M phases) of each cell line and 3-BrPA treated cells. (TIF) [file pone.0132285.s002.tif]

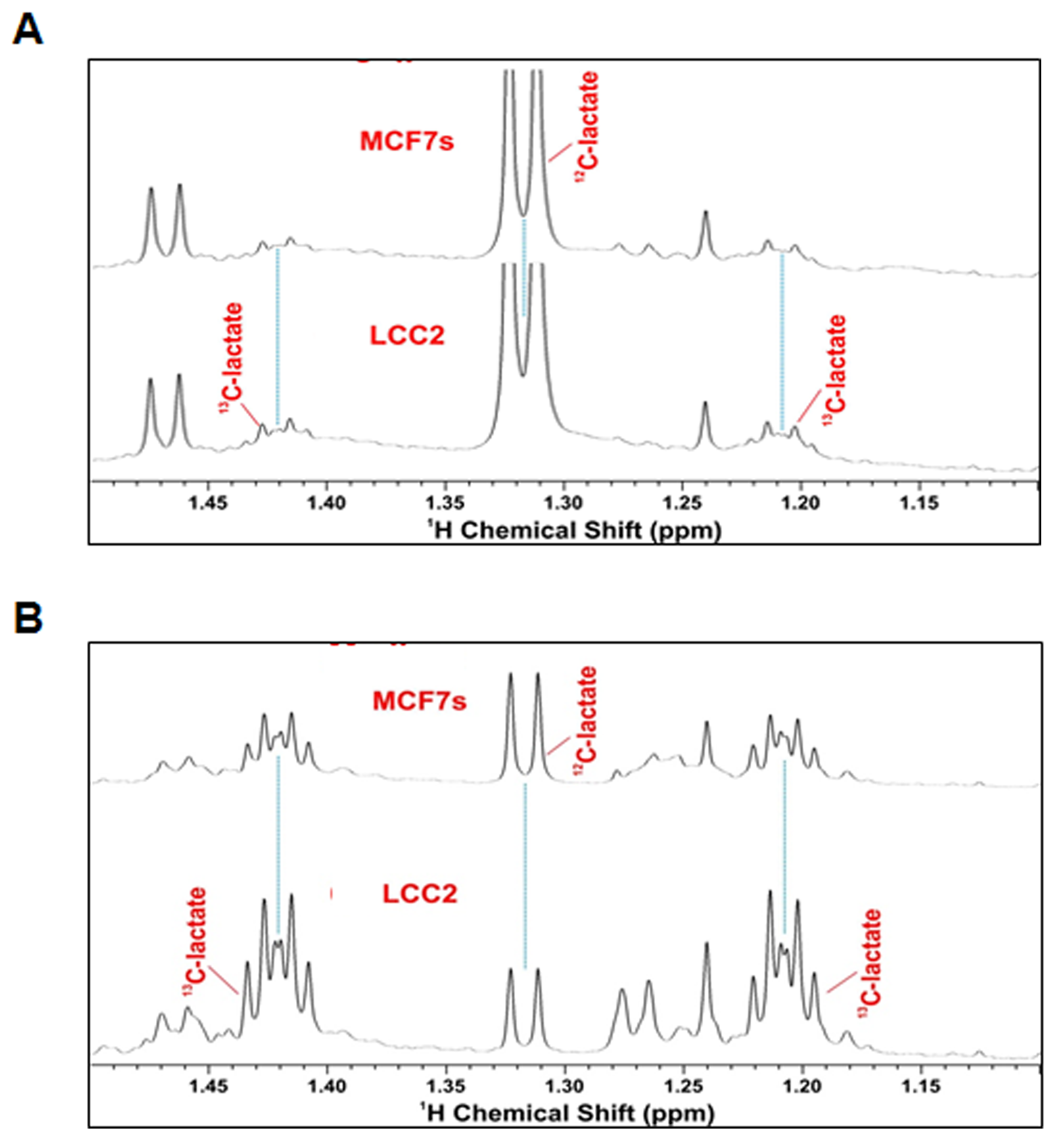

Supplement: S3 Fig — (TIF) [file pone.0132285.s003.tif]

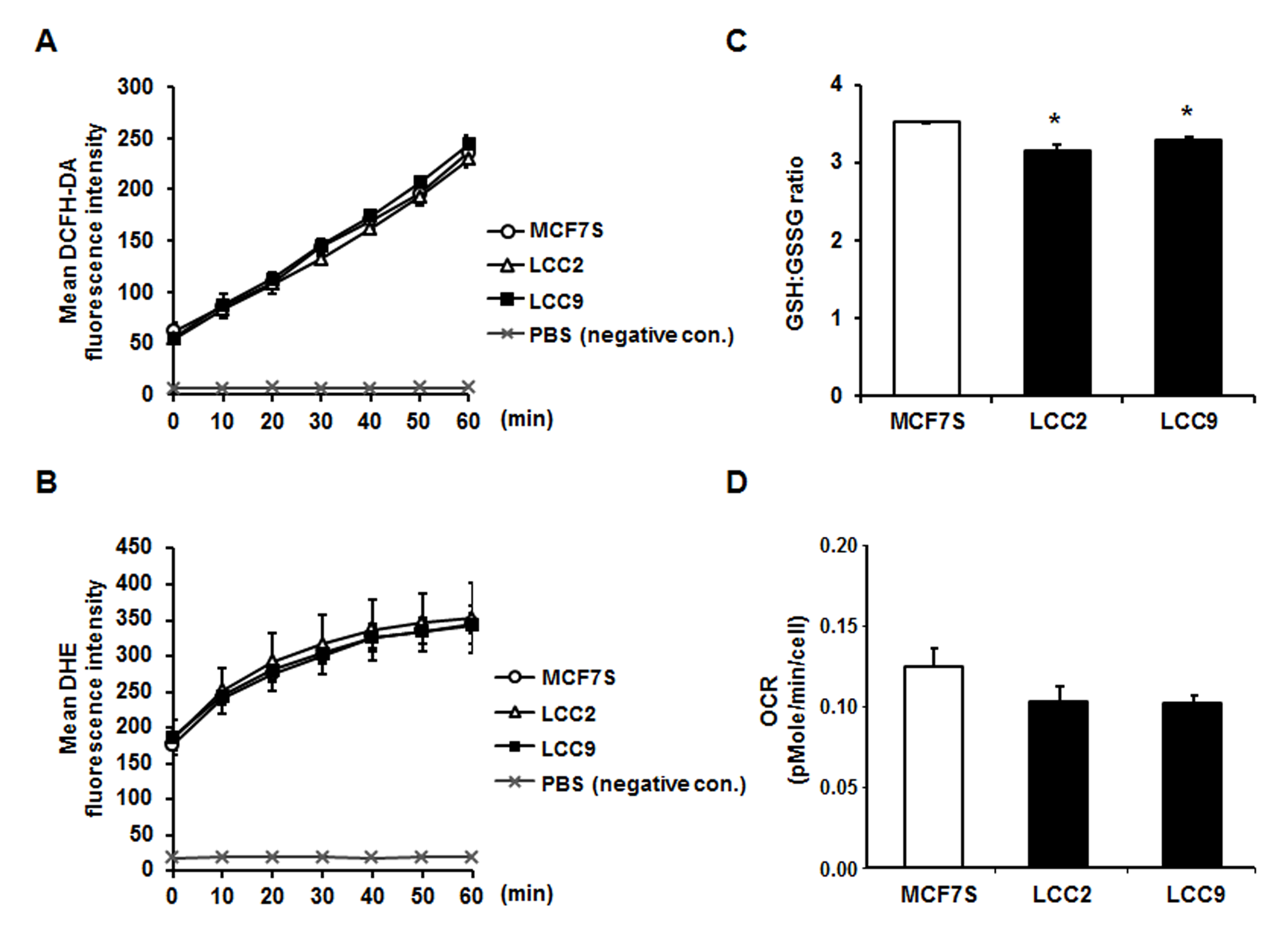

Supplement: S4 Fig — (A–B) Harvested cells were treated with DCFH-DA (50 μg/ml) or DHE (10 μg/ml) for the measurement of intracellular hydrogen peroxide and superoxide levels, respectively. The detection was performed by time-kinetics over 1 h at 10 min intervals. DCFH-DA- or DHE-treated 1× PBS was used as a negative control. (C) The concentrations of intracellular GSH and GSSG were measured and the GSH:GSSG ratio was calculated in MCF7S, LCC2, and LCC9 cells. *, P < 0.01. (D) Oxygen consumption rate was measured in three cell lines. The data are the mean ± SD of 3 independent experiments. (TIF) [file pone.0132285.s004.tif]
