# Supplementary material for: Inhibition of Aerobic Glycolysis Represses Akt/mTOR/HIF-1α Axis and Restores Tamoxifen Sensitivity in Antiestrogen-Resistant Breast Cancer Cells
Source: PLoS One. 2015 Jul 9;10(7):e0132285. doi: 10.1371/journal.pone.0132285 (PMC4497721; doi:10.1371/journal.pone.0132285)
Supplement: S1 File — (PDF) [file pone.0132285.s005.pdf]

Supporting Information

Inhibition of aerobic glycolysis represses Akt/mTOR/HIF-1α axis and restores tamoxifen sensitivity in antiestrogen-resistant breast cancer cells

Yu Mi Woo[1], Yubin Shin[1], Eun Ji Lee[1], Sunyoung Lee[1], Seung Hun Jeong[2], Hyun Kyung Kong[1], Eun Young Park[1], Hyoung Kyu Kim[2], Jin Han[2], Minsun Chang[3,*], and Jong-Hoon Park[1,*]

[1]Department of Life Systems, Sookmyung Women's University, 52 Hyochangwon Road, Yongsan-gu, Seoul 140-742, Republic of Korea; [2]National Research Laboratory for Mitochondrial Signaling Laboratory, Cardiovascular and Metabolic Disease Center, Department of Physiology, College of Medicine, Department of Health Sciences and Technology, Graduate School, Inje University, Gaegume 2 dong, Busanjin-gu, 633-165 Busan, [3]Department of Medical and Pharmaceutical Sciences, Sookmyung Women's University, 52 Hyochangwon Road, Yongsan-gu, Seoul 140-742, Republic of Korea, Korea

Supplementary materials and methods

Sulforhodamine B assay

A quantitative sulforhodamine B (SRB) colorimetric assay [1] was used to determine the inhibition of cellular growth by various compounds. Cells were plated at 1×10^3 cells/well in 96-well plates and grown for 24 h. The cells were treated with 4-OHT at 0, 2.5, 5, or 10 μM for 24 h. At the end of the incubation, the cells were fixed with 10% trichloracetic acid (1 h at 4°C), stained for 20 min at RT with 100 μl of a 0.4% w/v SRB solution (Sigma) in 1% acetic acid, and then quickly rinsed with 1% acetic acid. After air-drying, protein-bound dye was dissolved in 100 ml of 10 mM unbuffered Tris base at pH 10.5. The pink SRB was quantified by measuring the optical density at 540 nm. For each condition, the average cell density and the standard deviation were calculated, from the data points corresponding to the 6 wells.

Cell cycle analysis

The cell cycle analysis was performed by flow cytometry. Cells ($1x10^6$) were harvested, washed twice in PBS, and fixed in 70% ethanol at 4°C overnight. The fixed cells were stained with propidium iodide (PI)/RNase staining buffer (Becton Dickinson, 550825). Stained samples were analyzed by a FACS Canto II and the percentage of cells in each cell cycle phase was determined using FlowJo software. All samples were measured in triplicates with three different cell lines showed similar results.

Supplementary materials and methods

Measurement of reactive oxygen species

Reactive oxygen species (ROS) production was determined using 2′,7′-dichlorodihydrofluorescein diacetate (DCFH-DA) and dihydroethidium (DHE), which can be used to preferentially detect hydrogen peroxide and superoxide, respectively [2]. Cells were incubated in a 96-well plate at 2×10^5 cells/well with the addition of DCFH-DA (50 μg/ml) and DHE (10 μg/ml), respectively. Intracellular ROS levels were measured by monitoring fluorescence generated from the product via oxidation of either DCFH-DA or DHE at an excitation wavelength of 485 nm and emission wavelength of 535 nm for the product from DCFH-DA and an excitation wavelength of 488 nm and emission wavelength of 605 nm for the product from DHE during a 1-h reaction period [3].

Cellular glutathione concentration measurement

To measure the concentrations of intracellular reduced glutathione (GSH) and oxidized glutathione (GSSG), cells were detached by gentle trypsinization and one millions of cells were collected from each type of cell. The pellet was suspended with cold 5%(w/v) Metaphosphoric acid (Sigma Aldrich, 23927-5) and the suspension was centrifuged. The supernatant was transferred and lysed by sonication. Either total or oxidized glutathione was detected using Glutathione detection kit (Enzo, ADI-900-160) according to the manufacturer’s instruction.

Oxygen consumption measurements

XF24 analysis was performed as previously described [4]. Briefly, the XF24 cell culture plates (Part No. 100777-004, Seahorse Bioscience, USA) were shaped to each well of the 24-well plate. Then, the sensor cartridge was placed on top of the plate and incubated at 37°C without CO_2 overnight. The oxygen consumption rate (OCR) was measure by the XF24 analyzer and XF24 software. After measuring the OCR, the XF24 assay results were normalized to cell number. The cell number for each well was counted using a hemocytometer. Live cells were also counted and the XF assay results were normalized to cell number using XF assay software.

Supporting references

1. Lowry OH, Rosebrough NJ, Farr AL, Randall RJ (1951) Protein measurement with the Folin phenol reagent. J Biol Chem 193: 265-275.

2. Rothe G, Valet G (1990) Flow cytometric analysis of respiratory burst activity in phagocytes with hydroethidine and 2',7'-dichlorofluorescin. J Leukoc Biol 47: 440-448.

3. Jung MK, Song HK, Kim KE, Hur DY, Kim T, et al. (2006) IL-18 enhances the migration ability of murine melanoma cells through the generation of ROI and the MAPK pathway. Immunol Lett 107: 125-130.

4. Qian W, Van Houten B (2010) Alterations in bioenergetics due to changes in mitochondrial DNA copy number. Methods 51: 452-457.
